# Mini-Review: Antifouling Natural Products from Marine Microorganisms and Their Synthetic Analogs

**DOI:** 10.3390/md15090266

**Published:** 2017-08-28

**Authors:** Kai-Ling Wang, Ze-Hong Wu, Yu Wang, Chang-Yun Wang, Ying Xu

**Affiliations:** 1 Shenzhen Key Laboratory of Marine Bioresource & Eco-environmental Science, College of Life Sciences and Oceanography, Shenzhen University, Shenzhen 518060, China; kailingw@163.com (K.-L.W.); 13602652154@163.com (Y.W.); 2 Key Laboratory of Marine Drugs, Ministry of Education of China, School of Medicine and Pharmacy, Ocean University of China, Qingdao 266003, China; 3 Laboratory for Marine Drugs and Bioproducts, Qingdao National Laboratory for Marine Science and Technology, Qingdao 266200, China; 4 The Eighth Affiliated Hospital, Sun Yat-sen University, Shenzhen 518033, China; wuzehong922@126.com; 5 Integrated Chinese and Western Medicine Postdoctoral research station, Jinan University, Guangzhou 510632, China; 6 Institute of Evolution & Marine Biodiversity, Ocean University of China, Qingdao 266003, China

**Keywords:** antifouling, biofouling, marine microorganisms, marine natural products

## Abstract

Biofouling causes huge economic loss and generates serious ecological issues worldwide. Marine coatings incorporated with antifouling (AF) compounds are the most common practices to prevent biofouling. With a ban of organotins and an increase in the restrictions regarding the use of other AF alternatives, exploring effective and environmentally friendly AF compounds has become an urgent demand for marine coating industries. Marine microorganisms, which have the largest biodiversity, represent a rich and important source of bioactive compounds and have many medical and industrial applications. This review summarizes 89 natural products from marine microorganisms and 13 of their synthetic analogs with AF EC_50_ values ≤ 25 μg/mL from 1995 (the first report about marine microorganism-derived AF compounds) to April 2017. Some compounds with the EC_50_ values < 5 μg/mL and LC_50_/EC_50_ ratios > 50 are highlighted as potential AF compounds, and the preliminary analysis of structure-relationship (SAR) of these compounds is also discussed briefly. In the last part, current challenges and future research perspectives are proposed based on opinions from many previous reviews. To provide clear guidance for the readers, the AF compounds from microorganisms and their synthetic analogs in this review are categorized into ten types, including fatty acids, lactones, terpenes, steroids, benzenoids, phenyl ethers, polyketides, alkaloids, nucleosides and peptides. In addition to the major AF compounds which targets macro-foulers, this review also includes compounds with antibiofilm activity since micro-foulers also contribute significantly to the biofouling communities.

## 1. Introduction

Biofouling is defined as the undesirable colonization of submerged man-made surfaces by fouling organisms, including micro-organisms such as bacteria, algae and protozoa, and macro-organisms such as barnacles, bryozoans and tubeworms [1,2]. These fouling organisms not only cause huge material and economic loss in marine operations [3,4,5], but also create a series of environmental problems such as the spread of invasive species [6,7]. Coating the substrata with antifouling (AF) paints containing antifoulants or AF compounds is the most commonly used strategy to prevent marine biofouling. However, due to their toxicities toward non-target organisms, some AF compounds have raised many environmental issues and consequently led to increasing regulation of their usage [8,9,10]. For instance, the organotin compounds have been prohibited worldwide by the International Maritime Organization (IMO) since September 2008. Moreover, some alternative AF biocides of tributyltin (TBT), such as Irgarol 1051 and diuron, have been banned by many European countries in recent years because of the increasing evidence of their environmental risks [10,11]. Thus, there is an urgent demand for search of novel AF compounds without causing environmental issues.

Marine natural products are a potential source for the discovery of AF compounds. Over the last several decades, many compounds with AF EC_50_ values < 25.0 μg/mL (a standard established by the U.S. Navy program, actual EC_50_ values should be less than 5 μg/mL when selecting candidate AF compounds) have been isolated from seaweeds and marine invertebrates [12,13,14,15,16,17]. However, a major difficulty involved in commercialization of these active compounds for the marine coating industries is the supply issue. In contrast, marine microorganisms have recently attracted greater attention due to a number of benefits for industries, including the possibility of supplying large amounts of compounds through fermentation and genetic modification of the source organisms, as well as the ability of resource regeneration [18,19,20]. Indeed, many AF metabolites produced by marine microorganisms have been demonstrated as effective inhibitors of biofouling organisms, and some of these active substances have been considered as low- or even non-toxic antifoulants due to their high LC_50_/EC_50_ ratios (a compound with LC_50_/EC_50_ ratio > 15 is considered as a nontoxic antifoulant, but a much higher therapeutic ratio > 50 should be used when selecting candidate AF compounds) [15].

Although AF compounds have long been discovered from marine invertebrates, it was not until 1995 that the first publication describing AF compounds from marine microorganisms appeared [13,21]. In this paper, the AF metabolites were identified from the culture broth of a marine sponge *Halichondria okada*-associated bacterium *Alteromonas* sp. KK10304 [21]. Hence, this review describes 89 selected AF metabolites (EC_50_ values < 25.0 μg/mL) isolated from marine microorganisms during the period between 1995 and April 2017. Also included are 13 synthetic analogs of these AF natural products. All the compounds in this review, except the ones with antibiofilm activity, are sequentially introduced based on their chemical structures, including fatty acids, lactones, terpenes, steroids, benzenoids, phenyl ethers, polyketides, alkaloids, nucleosides and peptides. Potentials and challenges are also discussed with respect to the development of environmentally benign AF paints using natural products isolated from marine microorganisms.

## 2. Fatty Acids and Lactones

**Figure 1:**
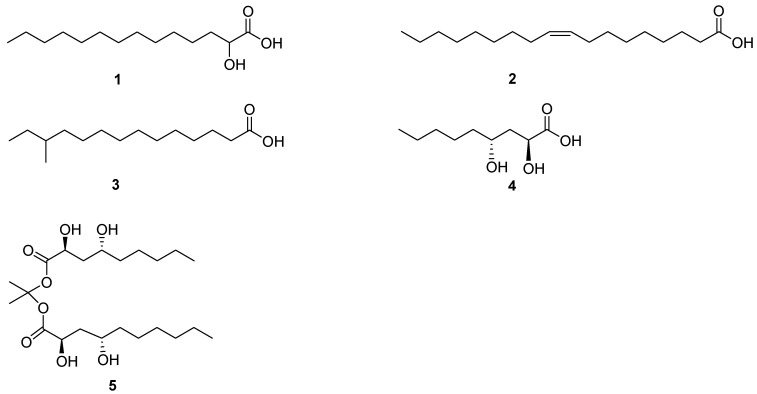
Chemical Structures of Fatty Acids **1**–**5**.

Two fatty acids, 2-hydroxymyristic acid (HMA) (**1**) and cis-9-oleic acid (COA) (**2**) (Figure 1), were isolated from the chloroform extract of a marine bacterium *Shewanella oneidensis* SCH0402 and blocked the germination of green algae *Ulva pertusa* spores completely at concentrations of 10 and 100 μg/mL, respectively. More significantly, no attachment of micro-fouling or macro-fouling organisms occurred on the surface of panels treated with 10% (*w*/*w*) HMA or COA after being immersed for 1.5 years in seawater, suggesting that this class of metabolites might have great value in commercial application [22]. Subsequently, AF bioassay-guided isolation afforded an HMA-like homolog, 12-methyltetradecanoid acid (12-MTA) (**3**) (Figure 1), derived from a deep-sea actinomycete *Streptomyces* sp. This compound showed strong AF activity against the polychaete *Hydroides elegans* larvae with an EC_50_ value of 0.6 μg/mL, while its toxicity was very low (LC_50_/EC_50_ ratio > 133.5). Studies on AF mechanism of 12-MTA against *H. elegans* revealed that the fatty acid mainly inhibited larval settlement through the down-regulation of the GTPase-activating gene and up-regulation of the ATP synthase gene [23]. (3*R*,5*S*)-3,5-dihydroxydecanoic acid (**4**) and a novel ester aureobasidin (**5**) (Figure 1) with an unusual 4,6-dihydroxydecanoic acid residue, obtained from the fermentation broth of a marine-derived fungus *Aureobasidium* sp., were demonstrated to display larval inhibition against the attachment of *B. amphitrite*, but the detailed data are not available [24].

**Figure 2:**
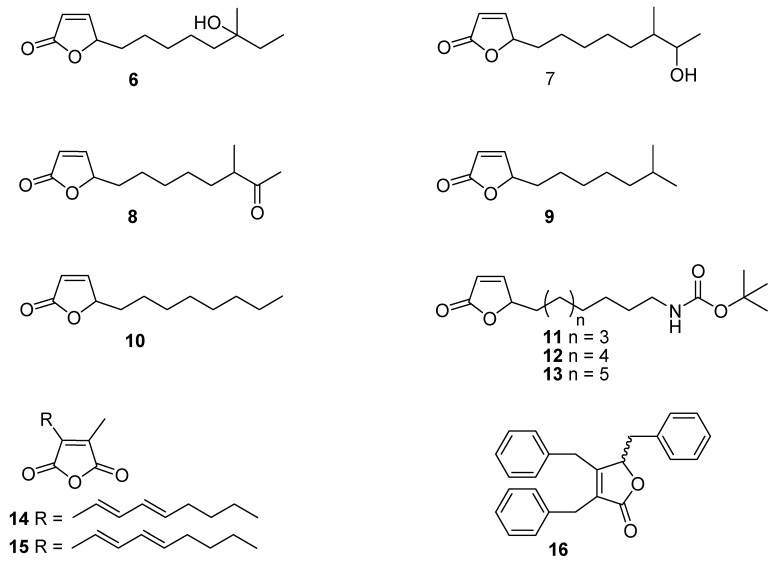
Chemical Structures of Lactones **6**–**16**.

Five structurally similar butenolides (**6**–**10**) (Figure 2) were isolated from the crude extract of a deep-sea-derived *Streptomyces albidoflavus* UST040711-291, among which **6**–**8** showed moderate AF activities against the larval settlement of *B. amphitrite* with the EC_50_ values of 14.81, 9.65 and 8.67 μg/mL, respectively [25]. Another butenolide compound, 10-methylundec-3-en-4-olide (**9**), isolated from the North Sea *Streptomyces* sp. strain GWS-BW-H5, was also inhibitory to *B. amphitrite* larvae with an EC_50_ value of 4.82 μg/mL [25,26]. Based on the preliminary analysis of the structure–activity relationship (SAR), the 2-furanone ring was suspected to be the functional group responsible for AF activities of these compounds, and the alkyl side-chain might affect their bioactivities by varying the lipophilicity of these compounds. A series of butenolide derivatives were then synthesized, among which compounds **10**–**13** (Figure 2) were highly active in inhibiting the larval settlement of *B. amphitrite* with the EC_50_ values of 0.518, 0.663, 0.722 and 0.827 μg/mL, respectively, while their toxicity towards the *B. amphitrite* cyprids was very low (LC_50_/EC_50_ ratios > 97, 61, 73 and 63, respectively) [27]. In addition, compound **10** also displayed strong and non-toxic AF activity against the larval settlement of *B. neritina* and *H. elegans* with the EC_50_ values of 0.199 and 0.0168 μg/mL and LC_50_/EC_50_ ratios higher than 250 and 119, respectively, indicating its broad-spectrum AF effects. Indeed, compound **10** exhibited excellent AF activity at a concentration of 5% (*w*/*w*) in field testing [25]. Interestingly, Hong and Cho [28] also isolated two compounds **14** and **15** (Figure 2) belonging to the butenolide class from a seaweed epibiotic bacterium *Streptomyces violaceoruber* SCH-09. Both compounds displayed significant inhibition against the seaweed *U. pertusa*, the diatom *Naviculaannexa*, and the mussel *Mytilus edulis* with the EC_50_ values ranging from 0.02 to 0.1 μg/mL, whereas these two compounds showed high therapeutic ratios (LC_50_/EC_50_ ratios > 92) for the three organisms tested. As mentioned above, butenolides may be considered as promising candidate antifoulants due to their simple structures, strong bioactivities in field assays, and non-toxicity towards the larvae of representative fouling organisms. Recently, Zhang et al. [29] and Qian et al. [30] reported the larvae of *B. amphitrite* and *B. neritina* responded to this class of butenolides by modulating their energy- and stress-related proteins. Another lactone with 2-furanone ring, maculalactone A (**16**) (Figure 2), was the most abundant secondary metabolite from a marine cyanobacterium *Kyrtuthrix maculans*, which showed a toxic effect on the naupliar larvae of the barnacles *B. amphitrite* , *Tetraclita japonica* and *Ibla cumingii* with the LC_50_ values ranging from 1.1 to 5.2 μg/mL. In the preliminary field investigation, this compound also showed some inhibitory effects on marine foulers, especially against the bivalves, at both concentrations: 0.1% and 1% (*w*/*w*) in a marine environment [31].

## 3. Terpenes and Steroids

**Figure 3:**
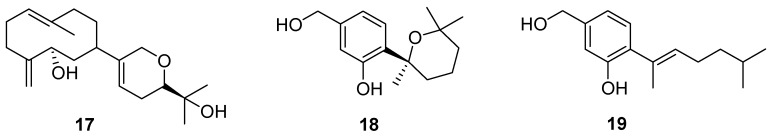
Chemical Structures of Terpenes **17**–**19**.

Lobocompactol (**17**) (Figure 3), an active AF diterpene, was isolated from a marine-derived actinomycete *Streptomyces cinnabarinus* PK209 and exhibited significant AF activity against the macro-alga *U*. *pertusa* and the diatom *N. annexa* (EC_50_ values of 0.18 and 0.43 μg/mL, respectively) with a large therapeutic ratio (LC_50_/EC_50_ ratios of 46.2 and 71.6, respectively), indicating that it might be a promising candidate as a nontoxic antifoulant targeting algae specifically. Lobocompactol also inhibited the growth of the fouling bacteria *Pseudomonas aeruginosa* KNP-5 and *Pseudomonas* sp. KNP-8 with the MIC values of 66 μg/mL and 112 μg/mL, respectively [32]. Four bisabolane-type sesquiterpenoids were isolated from the fungus *Aspergillus* sp. associated with the sponge *Xestospongia testudinaria*. Among them, compound **18** (Figure 3) could inhibit the larval settlement of *B. amphitrite* completely at the concentration of 25.0 μg/mL while compound **19** (Figure 3) was highly toxic to the larvae of *B. amphitrite* at this concentration [33].

**Figure 4:**
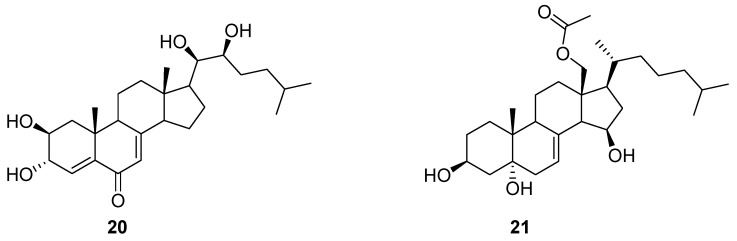
Chemical Structures of Steroids **20**–**21**.

Two steroids **20** and **21** (Figure 4), obtained from the crude extract of a red alga epiphyte filamentous bacterium *Leucothrix mucor*, were found to inhibit the spore settlement of *U*. *pertusa* zoospores (EC_50_ values of 1.2 and 2.1 μg/mL, respectively) and the growth of diatom (EC_50_ values of 5.2 and 7.5 μg/mL, respectively). Both compounds also inhibited the attachment of the biofilm-forming bacterial strains *P. aeruginosa* KNP-3 (MIC values of 32 and 56 μg/mL, respectively) and *Alteromonas* sp. KNS-8 (MIC values of 66 and 90 μg/mL, respectively) [34].

## 4. Benzenoids and Phenyl Ethers

**Figure 5:**
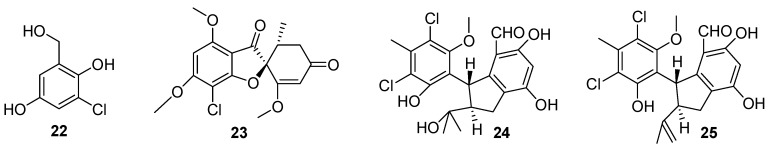
Chemical Structures of Benzenoids **22**–**25**.

Until now, to our best knowledge, only four natural benzenoids with chlorine atom have been isolated from three marine fungal strains and displayed inhibitive effects on micro- or macro-foulers. The first recorded metabolite 3-chloro-2,5-dihydroxybenzyl alcohol (**22**) (Figure 5) was isolated from a marine fungus *Ampelomyces* sp. UST040128. This compound showed an inhibitive effect on the larval settlement of *B. amphitrite* and *H. elegans* with the EC_50_ values ranging from 3.192 to 3.812 μg/mL and from 0.672 to 0.78 μg/mL, respectively. The LC_50_ value of **22** was calculated to be 267 μg/mL, indicating its non-toxicity towards *B. amphitrite* cyprids. However, 5 μg/mL of **22** killed all *H. elegans* larvae, and the calculated LC_50_ value was 2.642 μg/mL. In addition, the results of antibacterial bioassay using a standard disc-diffusion method were encouraging. At the concentration of 50 μg per disc, **22** showed different levels of inhibitory effects to the growth of 15 bacterial strains that were either marine pathogens or inductive strains for the larval settlement of *H. elegans* with inhibition zones ranging from 6.50 to 0.50 mm in diameter [35]. Another halogenated benzenoid amibromdole (**23**) (Figure 5) was extracted from a soft coral-derived fungus *Sarcophyton* sp., and exhibited weak AF activity against *B. amphitrite* larvae with an EC_50_ value of 16.70 μg/mL [36]. The final two compounds, (±)-pestalachlorides E (**24**) and F (**25**) (Figure 5), were isolated from a marine-derived fungal strain *Pestalotiopsis* ZJ-2009-7-6. Both of them showed potent AF activity against the larval settlement of *B. amphitrite* with the EC_50_ values of 1.65 and 0.55 μg/mL, respectively, meanwhile demonstrating no toxicity with LC_50_/EC_50_ ratios > 30.3 and 18.2, respectively [36].

**Figure 6:**
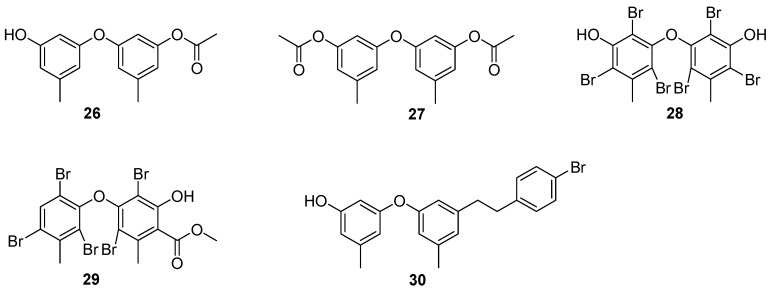
Chemical Structures of Phenyl Ethers **26**–**30**.

Six phenyl ethers were isolated as characteristic secondary metabolites of a marine-derived fungus *Aspergillus* sp. XS-20090066. These compounds, together with their seven synthetic phenyl ether derivatives were evaluated for their AF activity using *B. amphitrite* larvae. All of these compounds showed moderate to strong AF activity with the EC_50_ values ≤ 14.11 μg/mL. It should be noted that the synthetic compounds **26**–**30** (Figure 6) exhibited strong AF activity with the EC_50_ values ≤ 0.96 μg/mL, which was even lower than that of the positive control Sea-Nine 211^TM^ (EC_50_ value of 1.23 μg/mL), and their toxicity was also low with the LC_50_/EC_50_ ratios > 20. The investigation of SAR indicated that the ester group substitution at C-4 could increase AF activity for this class of natural phenyl ethers, and the introduction of bromine atoms played an important role in improving the AF activity of these synthetic derivatives [37].

## 5. Polyketides

**Figure 7:**
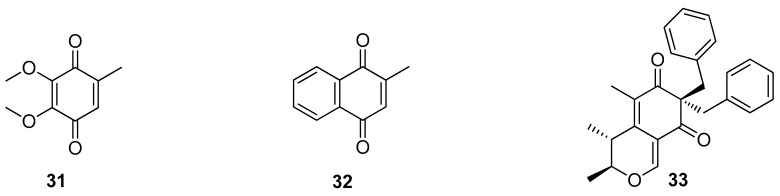
Chemical Structures of Polyketides **31**–**33**.

Polyketides are the largest family of secondary metabolites from microorganisms, and have provided a large number of compounds with structural and bioactive diversity, including macrolides, tetracyclics, anthraquinones, and polyethers. Two ubiquinones, ubiquinone-0 (**31**) and vitamin K3 (**32**) (Figure 7), were the earliest AF polyketides reported by Konya et al. [21]. Both compounds were isolated from the culture broth of *Alteromonas* sp. strain KK10304 associated with the sponge *Halichondria okadai* and could effectively inhibit the larval settlement of *B. amphitrite* at concentrations of 1.3 and 2.5 μg/mL, respectively, whereas they were very toxic to the barnacle *B. amphitrite* cyprids with the LC_30_ values of 5.2 and 2.5 μg/mL, respectively. Aspergilone A (**33**) (Figure 7), a novel benzylazaphilone derivative with an unprecedented carbon skeleton, was isolated from the gorgonian-derived fungus *Aspergillus* sp. This compound showed an EC_50_ value of 7.68 μg/mL against the settlement of *B. amphitrite* larvae. However, the symmetrical dimer of **70** with a unique methylene bridge, aspergilone B, displayed no AF activity [38].

**Figure 8:**
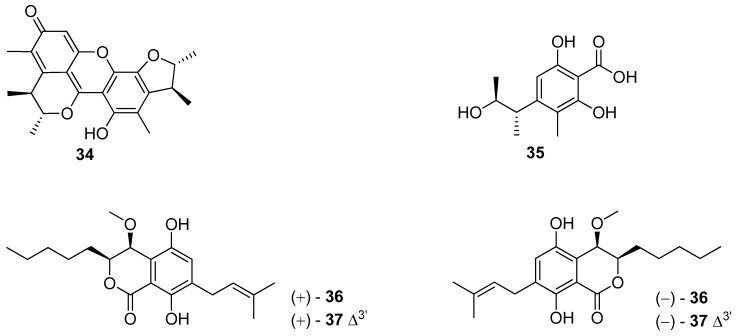
Chemical Structures of Polyketides **34**–**37**.

A marine-derived fungal strain *Xylariaceae* sp. SCSGAF0086 could produce dicitrinin A (**34**) and phenol A acid (**35**) (Figure 8). Dicitrinin A was more active with an EC_50_ value of 1.76 μg/mL than phenol A acid (EC_50_ value of 14.35 μg/mL) against the attachment of *B. neritina* larvae, and less toxic with LC_50_/EC_50_ ratio > 56 than phenol A acid (LC_50_/EC_50_ ratio > 15) [39]. Four pairs of rare dihydroisocoumarin derivatives, (±)-eurotiumides A–D, were isolated from a gorgonian-derived fungus *Eurotium* sp. XS-200900E6, and evaluated for AF activity against *B. amphitrite* larvae. All these compounds could inhibit larval settlement with the EC_50_ values ranging from 0.7–23.2 μg/mL, and (±)-eurotiumides B (±**36**) and (±)-eurotiumides D (±**37**) (Figure 8) were the most promising compounds with the EC_50_ values of 1.5, 0.7, 2.3, and 1.9 μg/mL, respectively, and they were not toxic to the cyprids (LC_50_/EC_50_ ratios > 20) [40].

**Figure 9:**
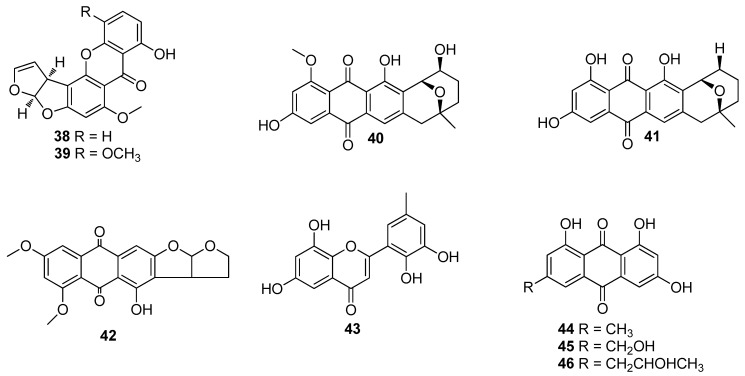
Chemical Structures of Anthraquinones and Xanthones **38**–**46**.

Anthraquinones and xanthones are the most prominent members of the polyketides family. However, the AF activity of this class of polyketides has been seldomly reported. Only eight metabolites **38**–**46** (Figure 9), mainly from marine fungi *Penicillium* sp. and *Aspergillus* sp. were demonstrated to have inhibitory effects on the larval settlement of *B. amphitrite* [37,41,42,43,44]. Compared with the AF activity of other six compounds, sterigmatocystin (**38**) and methoxysterigmatocystin (**39**), isolated from the fungal strains of the geneus *Aspergillus*, were much more active in inhibiting the larval settlement of *B. amphitrite* with the EC_50_ values < 0.125 μg/mL, and could paralyze the larvae at the effective concentration [42]. Another two anthraquinones averufin (**40**) and 8-*O*-methylnidurufin (**41**) from the fungus *Aspergillus* sp., together with one xanthone 6,8-di-*O*-methyl versiconol (**42**) from an unidentified mangrove fungus ZSUH-36, displayed weak AF activity against *B. amphitrite* larvae with the EC_50_ values of 2.03, 3.39, and 5.30 μg/mL, respectively [37,42,43,44]. Four anthraquinones **43**–**46** isolated from a gorgonian coral-derived fungus *Penicillium* sp. SCSGAF 0023 showed moderate AF activity against *B. amphitrite* larvae with the EC_50_values of 6.7, 6.1, 17.9, and 13.7 μg/mL, respectively [41].

**Figure 10:**
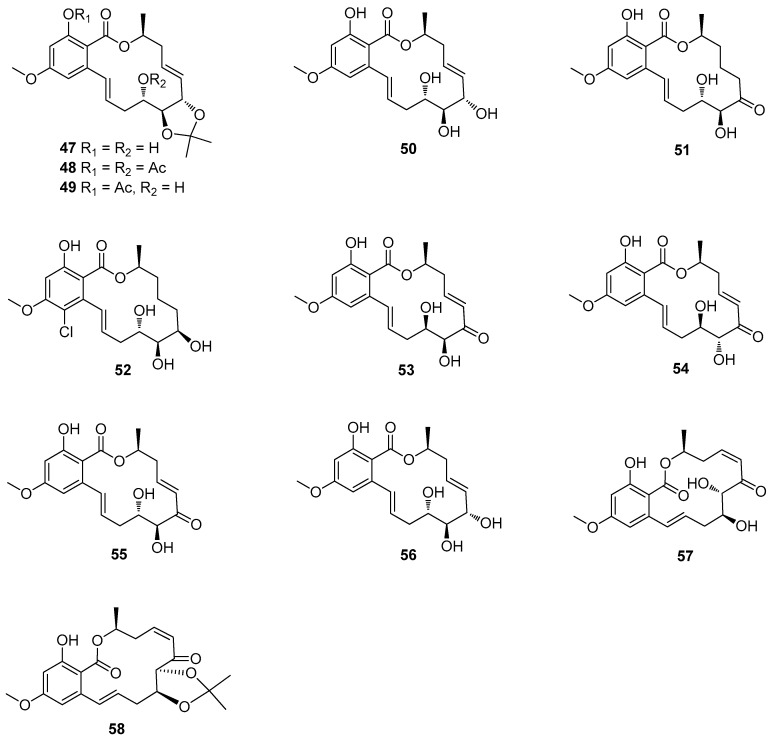
Chemical Structures of 14-membered Resorcylic Acid Lactones **47**–**58**.

Recently, a series of 14-membered resorcylic acid lactones, named cochliomycins, were isolated from the marine-derived fungi of the genus *Cochliobolus* [45,46]. This class of metabolites and their synthetic derivatives were demonstrated to have AF activity against the larval settlement of *B. amphitrite*. Shao et al. [45] firstly isolated seven cochliomycins from a gorgonian-derived fungus *C. lunatus*. Among them, compounds **47**–**52** (Figure 10) could inhibit the larval settlement with the EC_50_ values ranging from 1.2 to 17.9 μg/mL, among which cochliomycin A (**47**) showed the best AF activity with an EC_50_ value of 1.2 μg/mL and a LC_50_/EC_50_ ratio > 15. Analysis of SAR of **47**–**49** suggested the introduction of the acetonide moiety in **47** might improve its AF activity, and the hydroxy groups probably have a positive effect on the AF activity of these resorcylic acid lactones. Subsequently, cochliomycins D–F, along with eight known analogues, were isolated from the fungus *C. lunatus* associated with a sea anemone by Liu et al. [46]. In AF bioassays, compounds **53**–**58** (Figure 10) exhibited AF activity at nontoxic concentrations with the EC_50_ values ranging from 1.82 to 22.5 μg/mL, and LL-Z1640-2 (**55**) displayed the highest potency against *B. amphitrite* larvae with an EC_50_ value of 1.82 μg/mL. It was shown that the *cis*-enone moiety in **57** could significantly improve the EC_50_ value approximately 9-fold compared to that of **54** (EC_50_ value of 18.1 μg/mL) with *trans*-enone, indicating that the *cis*-enone functionality might contribute to the increase of AF activity. More interestingly, contrary to the enhancement of AF activity caused by the acetonide functionality of 14-membered resorcylic acid, the acetonide functionality led to the obvious decrease of AF activity for the enone resorcylic acid lactones. The above findings of SAR indicate that the AF activity of these 14-membered resorcylic acid lactones should be sensitive to subtle structural changes, such as the enone, the acetonide functionalities, and the hydroxy configurations. The preliminary AF mechanism revealed that the 14-membered resorcylic acid lactones mainly inhibited the larval settlement of *B. amphitrite* by stimulating the NO/cGMP pathway [47].

## 6. Alkaloids

**Figure 11:**
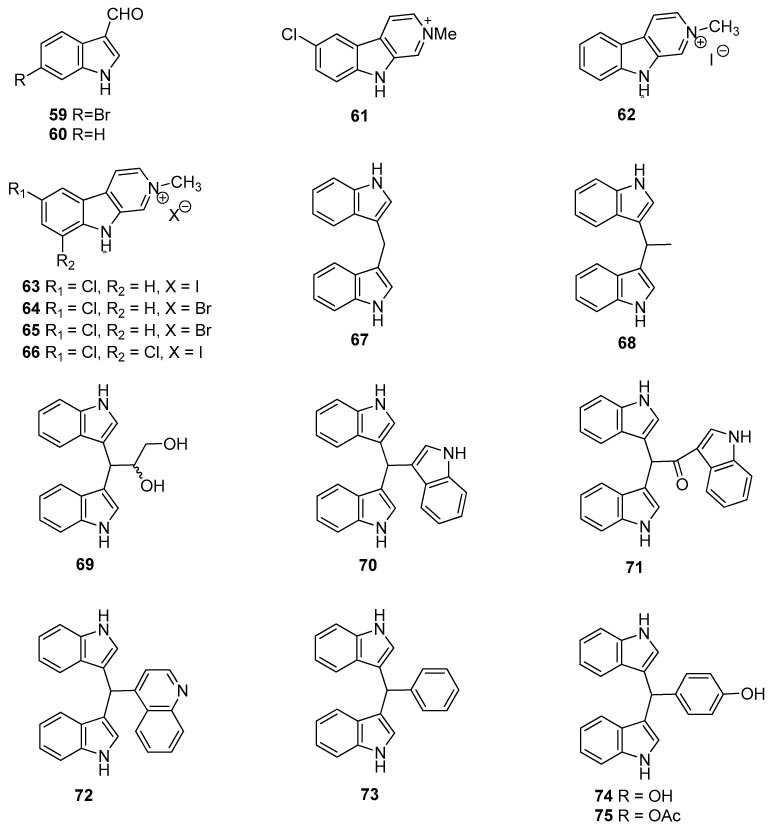
Chemical Structures of Alkaloids **59**–**75**.

In recent years, dozens of indole alkaloids were isolated from marine-derived bacteria, and some of them showed moderate to strong AF activities. A bacterial strain *Acinetobacter* sp*.* associated with the ascidian *Stomozoa murrayi* could produce two AF metabolites 6-bromoindole-3-carbaldehyde (**59**) and its debromo analog (**60**) (Figure 11) with the EC_50_ values of 5 and 28 μg/mL against *B. amphitrite* larvae, respectively [48]. Nostocarboline (**61**) (Figure 11), a carbonium alkaloid isolated from the freshwater cyanobacterium *Nostoc* sp., along with its five synthetic analogs **62**–**66** (Figure 11) were all active in inhibiting the growth of the toxic cyanobacterium *Microcystis aeruginosa* PCC7806, the nontoxic cyanobacterium *Synechococcus* PCC6911, and the green algae *Kirchneriella contorta* SAG 11.81. Among these six compounds, nostocarboline (**61**) showed strongest inhibitory activity against the growth of the cyanobacteria and algae with the MIC values of 0.22 μg/mL. The quaternary group and the replacement of the substituent on the 2-N atom appeared to be essential for increasing the anti-algal activity of this alkaloid class [49]. Eight bisindole products **67**–**74** (Figure 11) that belong to the di(1H-indol-3-yl)methane (DIM) family were isolated from a Red Sea ascidian-derived bacterial strain *Pseudovibrio denitrificans* UST4-50. All diindol-3-ylmethanes (DIMs) inhibited the larval settlement of *B. amphitrite* (EC_50_ values ranging from 0.63 to 5.68 μg/mL) with low toxicity (LC_50_ ratios > 15), of which DIM-Ph-4-OH (**74**) was the best AF compound with an EC_50_ value of 0.63 μg/mL. DIM (**67)** and DIM-Ph-4-OH also displayed significant AF activity against *B. neritina* larvae with the EC_50_ values of 0.62 and 0.42 μg/mL, respectively. Interestingly, the AF activity of DIM and DIM-Ph-4-OH against *B. amphitrite* cyprids was reversible. Moreover, DIM also showed comparable AF performance to the commercial AF biocide Sea-Nine 211™ in the field test over a period of five months, thus indicating that DIMs should be considered as promising candidates as useful antifoulants. Finally, in order to investigate the SAR of these DIMs, the acetylation of DIM-Ph-4-OH yielded the product DIM-Ph-4-OAc (**75**), which was less active as DIM-Ph-4-OH against the larval settlement of *B. amphitrite* and *B. neritina*. Based on the data of the AF activity of these natural metabolites and the acetylated derivative, it is suspected that the common moiety, di(1H-indol-3-yl)methylene, may serve as an important functional group for maintaining reversibly AF mechanism of DIMs, and that the phenolic hydroxyl group that presents at the Ph-C1′′′ of DIM-C1 will significantly enhance the AF activity of these bisindoles [50].

**Figure 12:**
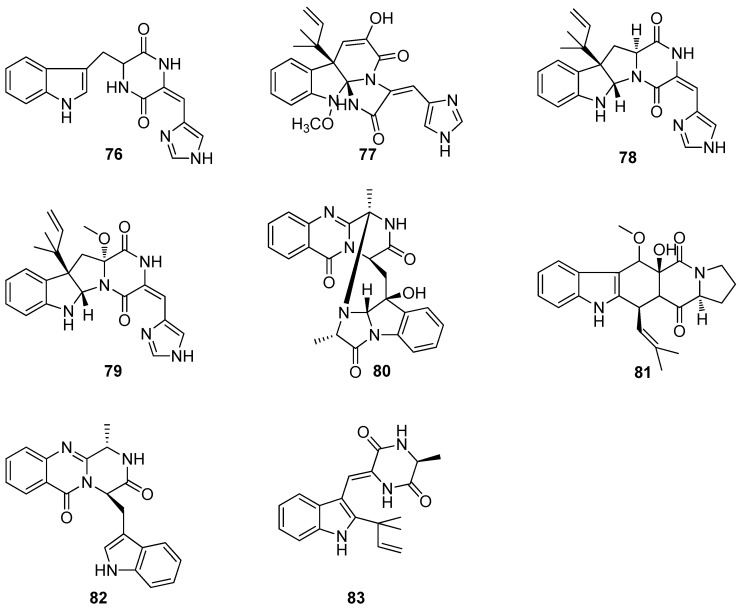
Chemical Structures of Alkaloids **76**–**83**.

In addition to marine bacteria, marine-derived fungal strains of *Aspergillus* sp. and *Penicillium* sp. have also been a rich source of similar structural indole alkaloids with AF activity. Compounds **76**–**79** and **80**–**83** (Figure 12), obtained from two marine fungi *Penicillium* sp. SCSIO 00258 and *Aspergillus sydowii* SCSIO 00305, were evaluated for their AF and antibacterial activities. All of the natural products **76**–**79** from the strain SCSIO 00258 exhibited antilarval settlement against *B. amphitrite* with the EC_50_ values of 12.3, 1.1, 6.2, and 17.5 μg/mL, respectively. Compounds **78** and **80**–**82** displayed AF activity against *B. neritina* larvae with the EC_50_ values of 2.1, 15.3, 8.4 and 8.2 μg/mL, respectively. Moreover, compounds **77** and **81** also showed modest antibacterial activity towards the larval settlement inducing bacterium *Micrococcus luteus* with MIC values of 200 and 300 μg/mL, respectively. Another indole alkaloid containing diketopiperazine, neoechinulin A (**83**), mainly isolated from marine-derived fungal strains of the genus *Aspergillus*, also inhibited the larval settlement of *B. amphitrite* with the EC_50_ value of 14.99 μg/mL [51].

**Figure 13:**
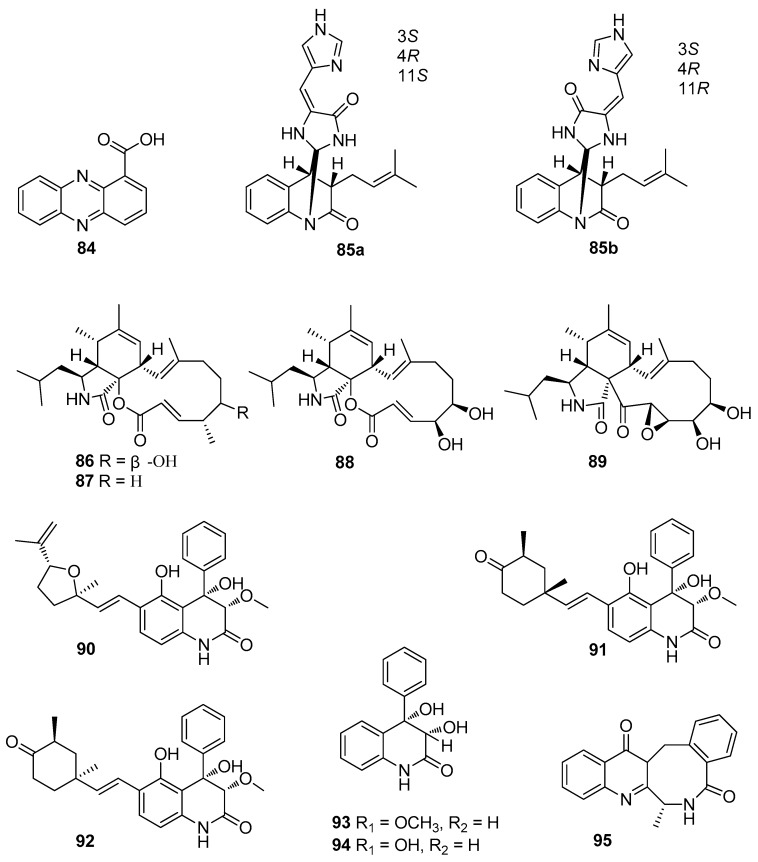
Chemical Structures of Alkaloids **84**–**95**.

At the same time, many other AF alkaloids were also obtained from marine bacteria and fungi. Screening of the chemical extracts from different bacteria incorporated into marine paints resulted in the discovery of an effective bacterium *Pseudomonas* sp. NUDMB50-11, which was isolated from the surface of Nudibranch. All petri dishes treated with the extracts of the broth culture of NUDMB50-11 showed a significant decrease in the settling frequency of barnacles and algal spores at a concentration of 20 mg/mL. The bioassay-guided fractionation and further purification of the crude extract of NUDMB50-11 led to the discovery of five metabolites, among which phenazine-1-carboxylic acid (**84**) (Figure 13) was the most active against all of the tested fouling bacteria with the MIC values ≤ 10 μg. Unfortunately, the detailed data of AF activities against barnacle larvae and fouling algae were not reported [52]. Penispirolloid A (**85a**/**b**) (Figure 13), a novel alkaloid possessing a unique spiro imidazolidinyl skeleton, was isolated from a halotolerant fungus *Penicillium* sp. OUCMDZ-776, though the absolute configuration of C-11 was undetermined. This compound displayed a significant AF activity against the larval settlement of *B. neritina* with an EC_50_ value of 2.40 μg/mL [53]. Ten cytochalasin alkaloids were isolated from the fermentation broth of a soft coral *Sarcophyton* sp.-derived fungus *A. elegans* ZJ-2008010, and they were evaluated for AF activity against *B. amphitrite* larvae. Four of them, determined as aspochalasins I (**86**), J (**87**), D (**88**), and H (**89**) (Figure 13), respectively, displayed strong to moderate AF activity with the EC_50_ values ranging from 2.59 to 15.44 μg/mL. Aspochalasin D, bearing an α,β-unsaturated ketone moiety, was found to be the most active compound, indicating that the electrophilic α,β-unsaturated carbonyl moiety and double-bond at C-19 and C-20 might be considerably important for increasing the AF activity of these cytochalasins. Additionally, this compound also exhibited broad spectrum of antibacterial activity, especially against pathogenic bacteria [54]. Six dihydroquinolin-2-one-containing alkaloids from a gorgonian coral-derived fungus *Scopulariopsis* sp. in the South China Sea could inhibit the larval settlement of *B. amphitrite*. Among these alkaloids, particularly important are compounds **90**–**94** (Figure 13), all of which were demonstrated to be highly active in antilarval settlement bioassay with the EC_50_ values of 0.007 pg/mL, 0.012 ng/mL, 0.001 ng/mL, 0.280 μg/mL and 0.219 μg/mL, respectively, while they were non-toxic with the LC_50_/EC_50_ ratio ranging from 57 to 1200, thus suggesting that this class of compounds are promising antifoulants [55]. Compound **95**, a benzodiazepine alkaloid isolated from a deep sea-derived fungus *Aspergillus westerdijkiae* SCSIO 05233, was reported as an inhibitor of *B. amphitrite* larvae with an EC_50_ value of 8.81 μg/mL [56].

## 7. Nucleosides and Peptides

**Figure 14:**
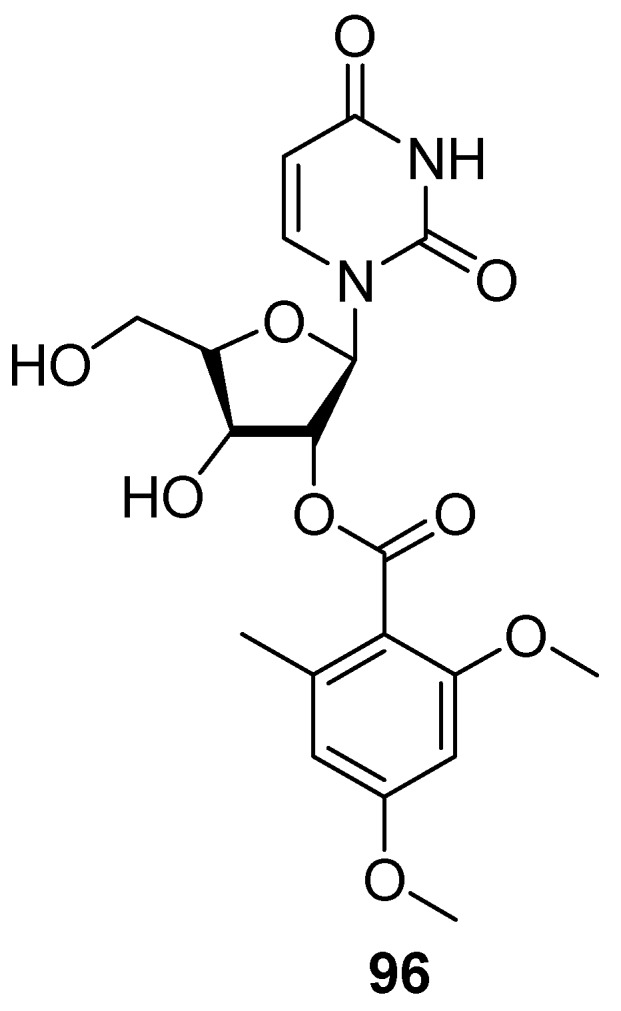
Chemical Structure of Nucleoside **96**.

AF nucleoside derivatives have been rarely reported. Only one nucleoside compound diacetylkipukasin E (**96**) (Figure 14), isolated from the fungus *Aspergillus versicolor* associated with a gorgonian *Dichotella gemmacea*, was found to have weak AF activity against *B. amphitrite* larvae with an EC_50_ value of 22.5 μg/mL [57].

**Figure 15:**
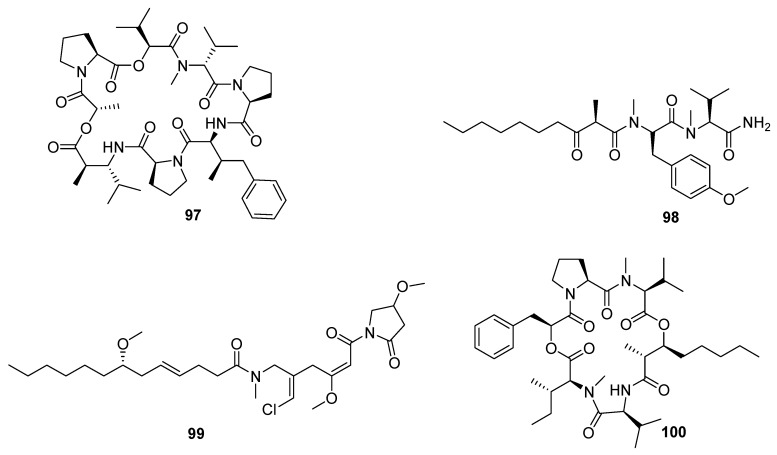
Chemical Structure of Peptides **97**–**100**.

Four secondary metabolites **97**–**100** (Figure 15) belonging to the mixed polyketide–polypeptide structural family were produced by a benthic filamentous marine cyanobacterium *Lyngbya majuscula* and they showed strong to moderate anti-larval settlement activity against *B. amphitrite* with the EC_50_ values of 0.003, 0.54, 2.6, and 10.6 μg/mL, respectively. In addition, it was noteworthy that the most active compound, dolastatin 16 (**97**), appeared to be non-toxic to the *B. amphitrite* cyprids with a LC_50_/EC_50_ ratio > 6000. More encouragingly, field testing of dolastatin 16 was conducted at concentrations of 10.0, 1.0, 0.1, and 0.01 μg/mL, respectively, and the barnacle counts of all dolastatin 16-treated plates were obviously lower than that of the negative controls after 4 weeks, suggesting its potential application as an antifoulant [58]. Although its total synthesis was challenging due to its complex structure, dolastatin 16 has been recently successfully synthesized by Casalme et al. [59]. The synthetic dolastatin 16 showed potent antilarval settlement of *B. amphitrite* similar to that of natural dolastatin 16, which further supported that dolastatin 16 might be explored as a candidate antifoulant.

**Figure 16:**
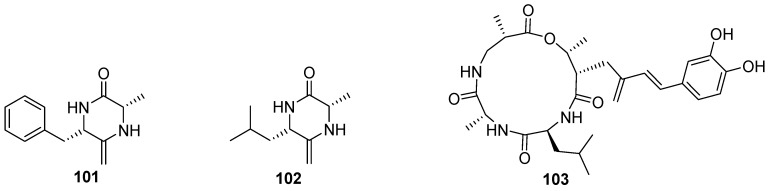
Chemical Structure of Diketopiperazines **101**–**103**.

Two diketopiperazines (DKPs), namely (6*S*,3*S*)-6-benzyl-3-methyl-2,5-diketopiper-azine (bmDKP) (**101**) and (6*S*,3*S*)-6-isobutyl-3-methyl-2,5-diketopiperazine (imDKP) (**102**) (Figure 16), were isolated from a seaweed *Undaria pinnatifida*–derived *Streptomyces praecox* 291-11. Both compounds inhibited zoospore settlement of the seaweed *U*. *pertusa* with the EC_50_ values of 2.2 and 3.1 μg/mL as well as the growth of the diotam *N. annexa* with the EC_50_ values of 0.8 and 1.1 μg/mL, respectively. Compared to the low therapeutic ratios of positive controls (LC_50_/EC_50_ ratios of 2 and 6 for the target organisms *U. pertusa* and *N. annexa*, respectively), bmDKP and imDKP showed higher therapeutic ratios (LC_50_/EC_50_ ratios of 17.7 and 21 to *U. pertusa*; 263 and 120.2 to *N. annexa*), indicating that these two compounds might be environmentally friendly antialgal compounds [60]. Aspergillipeptide C (**103**) (Figure 16), a cyclic tetrapeptide, was isolated from a culture broth of a gorgonian *Melitodes squamata*-derived fungus *Aspergillus* sp. SCSGAF 0076 and showed inhibitory attachment against *B. neritina* larvae with an EC_50_ value of 11 μg/mL and a LC_50_/EC_50_ ratio > 25 [61].

## 8. Enzymes

During the continuous investigation of enzymatic antifoulants, a variety of enzymes with AF activity have been obtained. Many patents of those promising enzymes have been applied [62]. According to Selvig et al. [63] and Polsenski & Leavitt [64], microorganism(s) alone or mixed with hydrolytic enzyme(s) could be incorporated into marine paints in order to test the AF activity of microorganisms or enzymes in field trials. A novel protease from a deep-sea derived bacterium *Pseudoalteromonasis sachenkonii* UST041101-043 showed strong inhibitive activity against the larval settlement of *B. neritina* with an EC_50_ value of 0.5 ng/mL. This enzyme also showed significant AF effects on the settlement of the barnacle *B. amphitrite* and the bryozoan *Schizoporella* sp. at a very low concentration of 100 ng/mL [65].

## 9. Biofilm Inhibitors

In the marine environment, a large number of various marine bacteria (especially those that induce settlement of fouling macro-foulers) communicate with each other by quorum sensing and form biofilms, which has an influence on marine macroorganism adhesion [66]. Therefore, it is promising to exploit those compounds with excellent anti-biofilm activity as potential antifoulants. Several marine invertebrates- or algae-derived metabolites and their synthetic analogs, including halogenated furanones, 2-amminoimidazole alkaloids, and flavonoids, have already been explored as candidate antifoulants [67,68,69,70,71]. However, only a handful of natural products derived from marine microbes with antibiofilm activity have been reported at this reported.

**Figure 17:**
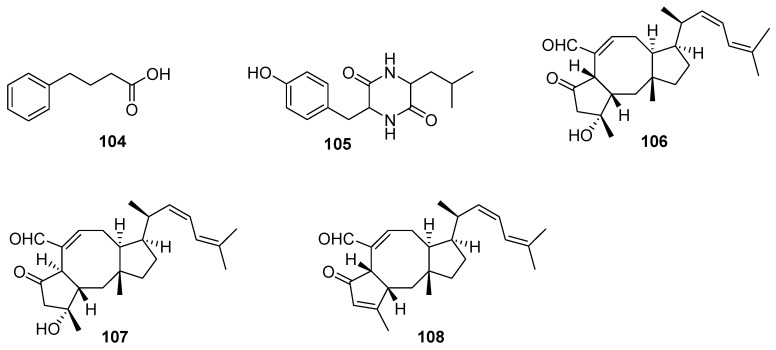
Chemical Structure of Biofilm Inhibitors **104**–**108**.

A glycolipid surfactant, composed of glucose and palmitic acid, was obtained from a tropical marine bacterium *Serratiamarcescens.* It prevented, to different degrees, the adhesion of the pathogens *Candida albicans* BH, and *P. aeruginosa* PAO1 as well as the marine biofouling bacterium *Bacillus pumilus* TiO1 at concentrations ranging from 0.125 to 25 μg/mL. More interestingly, this glycolipid could also efficiently disrupt the biofilm on glass surfacesper formed by the tested bacteria at a concentration of 50 μg/mL. The detailed structure of the glycolipid was yet to be determined [72]. 4-Phenylbutanoic acid (**104**) (Figure 17) was obtained from the crude extract of a marine bacterium *B. pumilus* S6-15 by Nithya et al. [73]. At concentrations ranging from 10 to 15 μg/mL, this compound showed potent biofilm inhibitory activity against all the 10 tested bacterial strains of Gram-positive and Gram-negative species. Scopel et al. [74] isolated a dipeptide, *cis*-*cyclo*(Leucyl-Tyrosyl) (**105**) (Figure 17) from a sponge-associated fungus *Penicillium* sp. F37, which was able to combat ~80% biofilm formation of a pathogenic bacterium *Staphylococcus epidermidis* at a concentration of 1.0 mg/mL without interfering with its growth. The search for new inhibitors of the biofilm formation of *Mycobacterium* species led to the discovery of three sesterterpenes named ophiobolin K (**106**), 6-epi-ophiobolin K (**107**), and 6-epi-ophiobolin G (**108**) (Figure 17) from the culture broth of a marine-derived fungal strain *Emericella variecolor*. All of these three compounds were capable of inhibiting biofilm formation of *Mycobacterium smegmatis* with the MIC values of 1.58, 24.97, and 6.23 μg/mL, respectively. In addition, ophiobolin K could restore the antimicrobial activity of isoniazid against the tested bacterium by inhibiting its biofilm formation. Ophiobolin K also showed antibiofilm activity against *Mycobacteriumbovis* BCG with a MIC value of 3.15 μg/mL [75].

## 10. Conclusions and Future Perspectives

Generally, when exploring candidate compounds for further development as AF products, the EC_50_ and LC_50_/EC_50_ values are often used as standards for the evaluation of the activity and toxicity of a given candidate AF compound. Although a compound with an EC_50_ value < 25 μg/mL and a LC_50_/EC_50_ ratio > 15 is often recommended as a non-toxic antifoulant, a much lower EC_50_ value and higher LC_50_/EC_50_ ratio is required when screening for candidate antifoulants. As pointed out by Qian et al. [15], only those small molecules which exhibit AF activities against a broad spectrum of biofoulers with an EC_50_ value < 5 μg/mL and a LC_50_/EC_50_ ratio > 50 should be considered. Meanwhile, the properties of the AF substances, such as solubility and stability, should also be given enough attention during selection. A total of 89 AF/anti-biofilm natural products from marine microorganisms and 13 of their chemical synthetic derivatives with EC_50_ value < 25 μg/mL are described in this review. Among them, 40 compounds are potent AF compounds with EC_50_ < 5 μg/mL against representative fouling organisms, such as fouling bacteria, algae, barnacle and bryozoan. Meanwhile, they had high LC_50_/EC_50_ ratios greater than 50. These data support that marine microorganisms could be a promising source for the discovery of highly effective and low- or non-toxic AF compounds.

There are a number of advantages using marine microorganisms as sources of antifoulants. First of all, as mentioned in this review, AF metabolites isolated from marine microorganisms are chemically diverse and unique, which makes them promising sources for the exploitation of novel candidate antifoulants. Second, having a large collection of marine invertebrates and algae to meet the supply demand of AF compounds leads to the concern of reducing biodiversity of the marine eco-environment. In contrast, if microbes are selected as a source for AF compounds, the supply issues could be solved by culturing the microorganisms and using genomic tools to identify the genes responsible for biosynthesis of the active metabolites. In addition, microbial strains of the same species can produce different bioactive metabolites under different temperatures, pH and nutrient conditions, which further enriches the chemical scope of AF compounds.

The major challenges for the development of AF coatings highlighted in reviews by Fusetani [14,16], Qian et al. [15,17], Dobretsov et al. [18,19], Maréchal and Hellio [76] and Satheesh et al. [20] remain in the current industrial application of those AF compounds. Therefore, several issues must be addressed in future research. Firstly, some AF compounds with complicated structures are very hard to synthesize’. A combination of the fermentation strategy of microorganisms’ genetic technologies and metabolomics is required to generate sufficient quantities of target compounds. Secondly, even though various compounds with strong activities against typical biofoulers produced by marine microorganisms have been obtained since 1995, there is little information on the field tests of these compounds. Therefore, it is necessary to evaluate highly effective compounds in field assays for the spectrum of their AF activity, which will provide critical data for the marine coating industries. Thirdly, with the increasing ecological problems of heavy metal and organotins-alternative biocides, the acute and chronic toxicity towards non-target organisms and degradation kinetics of potent AF agents in the marine ecosystem needs to be considered before commercialization. Especially for more complex compounds, some of them are not easy to break down in the marine environment, and may cause biological accumulation for marine organisms, which will induce long-term physiological impacts on the food chain. At the same time, it is very necessary that the toxicities of degradation products of those complex compounds should be evaluated to guide their intelligent usage. Moreover, the mechanisms of AF compounds against target fouling organisms remain poorly studied due to the difficulty of establishing a model-fouling organism in a laboratory for the examination of AF mechanisms using molecular tools. Over the past five years, a number of genes and proteins that may be involved in normal/drug-treated larval settlement of model fouling organisms, such as *B. amphitrite* [29,47,77,78,79], *B. neritina* [30,80], and *H. elegans* [23], have been identified through genomic and proteomic approaches. More efforts should be invested to decipher specific genes/proteins or pathways involved in the settlement/anti-settlement process, which will not only facilitate AF candidates to be introduced to the market but also help to identify molecular biomarkers for rapid screening of AF activity of marine natural products. In summary, the process for the registration (or “approval”) of potent antifoulants is time- and cost-consuming. The two organizations Australian Pesticides and Veterinary Medicines Authority (APVMA) and Australian Paint Approval Scheme (APAS) have well-developed systems for establishing specification requirements of antifouling paints, which include efficacy standards, antifouling classifications, available and/or relevant standards and specifications, antifouling test facilities, biocidal/non-biocidal systems, and a national antifouling standards and specifications working group [81]. Finally, the APVMA will disseminate the acceptable paints on its website, while the APAS will publish a “List of Approved Products”.

Last but not least, formation of marine biofilms has been increasingly recognized as an important factor in influencing fouling of marine macro-biofoulers [66,82,83,84], but exsisting knowledge of the interaction between biofilms and settling propagules and the molecular mechanism of anti-biofilm compounds is quite limited. Marine biofilms are perhaps much more serious than macro-fouling for the marine industry and for aquaculture, which helps settlement of macro-foulers. Yet so far, only several marine-derived antibiofilm compounds have been isolated and identified. Chemical studies of marine natural products will help to fill in the gaps in of this area.

Overall, it is well recognized that marine microorganisms could provide a promising source for AF compounds. With the improvement of cultivation and isolation methods and the employment of molecular tools to modify microbial strains, more isolates and novel AF compounds may be obtained, which will provide more candidates for the marine coating industries. However, there are still many obstacles restricting transformation of laboratory bioassay results to field applications. To meet these major challenges as mentioned in this review, the establishment of new bioassay systems with respect to diverse fouling organisms and systematic platforms for the evaluation of marine environmental risks, and explicit understanding of the bioactive mechanisms of the target compounds is desired for the market-demanded antifoulants. Without doubt, development of a candidate natural compound into a commercial product requires a large investment of time and cost. Therefore, a close collaboration between the coating industries and research laboratories might be a wise choice for the screening of effective and environmentally friendly antifoulants from marine microorganism-derived metabolites.
